# PPT1‐mediated plastidic phospho *enol* pyruvate import enhances fatty acid biosynthesis in sugar‐rich tissues

**DOI:** 10.1111/nph.71160

**Published:** 2026-04-07

**Authors:** Jiang Wang, Yi‐Hsuan Lin, Xueyi Xue, Gabriel Beuchat, Yaxin Li, Jiankun Li, Li‐Qing Chen

**Affiliations:** ^1^ Carl R. Woese Institute for Genomic Biology University of Illinois at Urbana‐Champaign Urbana IL 61801 USA; ^2^ Center for Advanced Bioenergy and Bioproducts Innovation, University of Illinois at Urbana‐Champaign Urbana IL 61801 USA; ^3^ Department of Plant Biology University of Illinois at Urbana‐Champaign Urbana IL 61801 USA

**Keywords:** *Arabidopsis thaliana*, biofuel, metabolic engineering, phospho*enol*pyruvate, PPT, SWEET

## Abstract

- Metabolic engineering of vegetative tissues for lipid production holds transformative potential for sustainable biofuels, yet achieving sufficient yields remains challenging. Here, we present a strategy to enhance fatty acid synthesis by redirecting cytosolic phospho*enol*pyruvate (PEP) into plastids through overexpression of the plastidial phospho*enol*pyruvate/phosphate translocator (*PPT1*) in vegetative tissues of *Arabidopsis thaliana*.
- Integrated metabolomic and transcriptomic analyses revealed that *AtPPT1* overexpression alleviated metabolite overaccumulation in high‐sugar tissues, consistent with enhanced carbon flux coordination between the cytosol and chloroplast. Notably, phosphofructokinase activity, a key step in glycolysis, was elevated, linking plastidic PEP import to increased glycolytic throughput.
- In Arabidopsis, overexpression of *AtPPT1* increased fatty acid content and lipid droplet accumulation in the sugar‐accumulating *sweet11;12;13* mutant, but not in wild‐type Col‐0.
- Together, these findings establish PEP redirection as an effective strategy to boost fatty acid and lipid production in sugar‐rich vegetative tissues and provide a complementary metabolic module for future lipid‐engineering efforts.

## Introduction

Bioenergy derived from feedstock crops presents a promising sustainable alternative to fossil fuels, offering the potential to reduce carbon emissions and mitigate climate change (Field *et al*., 2020). Currently, bio‐oil production relies primarily on oilseed crops such as soybean and canola, creating competition between fuel and food resources (Ambaye *et al*., 2021). By contrast, nonoil plants, particularly their vegetative tissues, naturally accumulate only limited levels of total fatty acids (TFA) with minimal storage lipids like triacylglycerol (TAG) (Durrett *et al*., 2008). To overcome this limitation, significant efforts have been directed toward metabolically engineering high‐biomass feedstocks to enhance oil production in their vegetative tissues (Hofvander *et al*., 2016; Vanhercke *et al*., 2019; Liang *et al*., 2023; Cao *et al*., 2023a, 2023b; Khan *et al*., 2025; Park *et al*., 2025). A prevailing strategy involves the coordinated overexpression of three key lipogenic factors: the transcription factor WRINKLED1 (WRI1), a master regulator of fatty acid biosynthesis (Cernac & Benning, 2004); DIACYLGLYCEROL ACYLTRANSFERASE1 (DGAT1), which catalyzes the key final step of TAG assembly (Lehner & Kuksis, 1996); and OLEOSIN1 (OLE1), a structural protein that stabilizes lipid droplets (LDs; Sadeghipour & Bhatla, 2002). Despite these advances, achieving sufficient yields requires the development of complementary engineering strategies to further boost lipid accumulation.

A critical metabolic node for enhancing fatty acid synthesis is the supply of phospho*enol*pyruvate (PEP) to plastids, where it serves as a precursor for both fatty acid and aromatic amino acid biosynthesis, with direct implications for chloroplast development and plant growth (Li *et al*., 1995; Streatfield *et al*., 1999; Tang *et al*., 2022). Plastidic PEP can be supplied through three potential routes: (1) plastid‐localized glycolysis via phosphoglyceromutase (PGyM) and enolase (ENO) (Prabhakar *et al*., 2009); (2) direct import from the cytosol via the PEP/phosphate translocator (PPT) (Fischer *et al*., 1997); or (3) conversion from pyruvate by pyruvate orthophosphate dikinase (PPDK) (Aoyagi & Bassham, 1984). Although plastidic pyruvate can be imported from the cytosol via bile acid : sodium symporter family protein 2 (BASS2) (Furumoto *et al*., 2011), it is not clear whether this route occurs at a rate sufficient for PEP synthesis through PPDK. Previous work in *Petunia hybrida* has demonstrated that cosilencing of plastidic ENO and PPT markedly reduces PEP supply and downstream metabolites such as shikimate, flavonoids, and aromatic amino acids, indicating that plastidic PEP import via PPT contributes significantly to carbon allocation in primary and secondary metabolism (Li *et al*., 2025). In Arabidopsis, two PPT isoforms exist, and the mutant of *PPT1*, also known as *Chla/b‐binding protein underexpressed 1* (*cue1*) (Li *et al*., 1995), causes a reticulate leaf phenotype and impaired chloroplast development. These defects are fully rescued by *PPT1* but only partially by *PPT2* or *PPDK* (Knappe *et al*., 2003; Voll *et al*., 2003), suggesting the physiological importance of PEP import.

Despite PPT's central role, the metabolic consequences of enhancing PPT activity remain poorly understood, particularly in vegetative tissues. Although overexpression of *BnaPPT1* in *Brassica napus* increased seed oil content, TFA levels at seed maturity were unchanged (Tang *et al*., 2022), suggesting that PPT‐mediated PEP import may not be limiting in lipid‐storing tissues. Notably, lipid‐storing tissues such as seeds possess a complete plastidial glycolytic pathway, enabling endogenous PEP production and potentially reducing reliance on cytosolic import (Fischer *et al*., 1997; Flügge *et al*., 2011). By contrast, plastidial glycolysis in plant leaves is less significant compared to the cytosol, rendering plastidial metabolism more dependent on cytosolic PEP supply. We therefore hypothesized that enhancing PPT activity would have a greater impact on carbon flux and fatty acid biosynthesis in nonlipid‐storing vegetative tissues than in seeds.

Given that PEP is a central metabolite required for both cytosolic and plastidial pathways, we further hypothesized that enhancing PEP import into plastids would stimulate fatty acid biosynthesis only when cytosolic carbon surplus is in excess, thereby avoiding depletion of precursors needed for essential cellular respiration. To test this hypothesis, we overexpressed *AtPPT1* in both wild‐type plants and a sugar‐hyperaccumulating genetic background (*sweet11;12;13*), providing a conceptual context to evaluate how cytosolic carbon availability influences the metabolic outcome of enhanced plastidic PEP transport.

## Materials and Methods

### Plant materials and growth conditions

*Arabidopsis* *thaliana*  (L.) Heynh (Col‐0) plants were grown at 22°C under a 16 h : 8 h, light : dark cycle with 100–150 μmol m^−2^ s^−1^ light. The *atsweet13* mutant (SALK_087791) was obtained from ABRC, and *atsweet11;12* mutants were previously described (Chen *et al*., 2012). Triple mutants (*atsweet11;12;13*) were generated by crossing and genotyped using primers P1–P6 (Supporting Information Table S1). Transgenic lines were generated via floral dip (Clough & Bent, 1998), with ≥ 16 T_1_ lines per construct and ≥ 3 T_2_ lines with strong fluorescence used for experiments. Plants were grown in PRO‐MIX potting soil, and mature leaf samples (third and fourth) were collected at 21 d post‐germination (DPG) unless otherwise noted.

### Constructs for Arabidopsis transformation

The CDS region of *AtPPT1* was synthesized (GenScript, Piscataway, NJ, USA) with synonymous mutations made to remove internal *Dra*III, *Bbs*I, and *Bsa*I restriction sites and was subcloned into pL0V to make pL0M‐SC1‐AtPPT1. Subsequently, pL1M‐F2‐p*35S‐AtPPT1‐eGFP‐tNOS* was assembled using the Golden Gate cloning protocol (Weber *et al*., 2011) before further assembling into a binary vector (pL2V‐1) with p*L1M‐R1‐p35S‐HYG‐tNOS*. The binary vector was incorporated into *Agrobacterium tumefaciens* strain *GV3101* before transforming into wild‐type Col‐0 and *atsweet11;12;13*.

### Fatty acids determination

The TFA profiles from *c*. 20 mg freeze‐dried samples were determined using a direct acid‐catalyzed transmethylation method, as previously reported (Wang *et al*., 2022).

### Soluble sugar determination

Soluble sugar components from *c*. 5 mg freeze‐dried samples were determined as previously reported (Wang *et al*., 2022) using a high‐performance liquid chromatography (HPLC) coupled with a refractive index detector (RID).

### Starch determination

Fresh mature leaf samples were collected at 21 DPG, and the starch quantification was performed using the anthrone method (Clegg, 1956).

### Phosphofructokinase (PFK) activity determination

The third and fourth leaves were collected at 21 DPG at the end of the day for PFK activity analysis. Extraction and measurement of PFK activity were performed using a PFK assay kit (MBS9719203, MyBioSource, CA, USA). Crude enzyme was extracted and used in a reaction mixture that catalyzes the conversion of fructose 6‐phosphate and ATP into fructose 1,6‐bisphosphate and ADP. Pyruvate kinase and lactate dehydrogenase were subsequently used to catalyze the oxidation of NADH to NAD^+^. PFK activity was determined by monitoring the decrease in NADH absorbance at 340 nm.

### Nile Red staining

LDs from Arabidopsis leaves were visualized via Nile Red staining as previously reported (Winichayakul *et al*., 2013) using confocal microscopy.

### Microscopy imaging

For GFP acquisition, images were taken using LSM 880 (Carl Zeiss, New York, NY, USA). Argon laser excitation wavelength and emission bandwidths were 488 nm (10% intensity) and 493–545 nm for GFP (gain 600), 633 nm (10% intensity) and 634–686 nm for Chl autofluorescence (gain 600), respectively. For Nile Red acquisition, images were taken using LSM 710 (Carl Zeiss). Argon laser excitation wavelength and emission bandwidths were 514 nm (10% intensity) and 557–599 nm for Nile Red (gain 800), 514 nm (10% intensity) and 619–697 nm for Chl autofluorescence (gain 350), respectively.

### RNA isolation and reverse transcription‐quantitative polymerase chain reaction (RT‐qPCR)

RNA isolation and RT‐qPCR were performed as previously described (Wang *et al*., 2022). Detailed qPCR primers were recorded in Table S1 (P7–P40). The expression values were normalized to *ACT8* for Arabidopsis in each repeat (Livak & Schmittgen, 2001). The analyses were based on three biological replicates.

### RNA‐seq analysis

The cDNA libraries were prepared from 1 μg RNA and were sequenced at the Roy J. Carver Biotechnology Center at the University of Illinois at Urbana‐Champaign. Fastq files were trimmed using Trimmomatic (Bolger *et al*., 2014) with a minimum length of 60. They were aligned to the Araport11 gene set (Cheng *et al*., 2017) with the STAR aligner program (Dobin *et al*., 2013) to produce BAM files, and these were converted to counts with the featcounts program (Liao *et al*., 2014).

Differentially expressed gene (DEG) analysis was conducted in RStudio using the deseq2 package from Bioconductor, with log_2_ fold change shrinkage performed using the ashr method to improve the stability of effect size estimates. Genes with an adjusted *P*‐value < 0.05 and an absolute log_2_ fold change ≥ 0.5 were considered significantly differentially expressed (Gentleman *et al*., 2004; Love *et al*., 2014; Stephens, 2017). PCAs were carried out on a log transformation of the count data using the plotPCA function of DESeq2 and ggplot2 (Wickham, 2016). For the matrix, only genes whose median expression in at least one treatment group was 5 Transcripts Per Million (TPM) or greater were included. Heatmaps were carried out on TPM data converted to *z*‐scores for each gene using the pheatmap package (Kolde, 2019). Kyoto Encyclopedia of Genes and Genomes (KEGG) and Gene Set Enrichment Analysis (GSEA) were performed using the OmicShare tools with default settings using a false discovery rate *q*‐value cutoff of 0.05 (http://www.omicshare.com/tools).

### LC‐MS and GC‐MS analysis

Metabolite analysis was performed by the Metabolomics Core Facility of the Roy J. Carver Biotechnology Center, University of Illinois Urbana‐Champaign. Samples were homogenized with Bead Mill 4 (Fischer Scientific, MA, USA), and compounds were partitioned in 1 ml of methanol : chloroform (2 : 3 v/v) (lipid analysis) and 1 ml of distilled water (glycolysis intermediates analysis) followed by centrifuging at 20 000 ***g*** for 15 min at 4°C. Supernatants were collected into separate tubes, evaporated, and resuspended in 100 μl of methanol : chloroform (2 : 3 v/v) or 20 mM ammonia acetate.

PEP, DHAP, G3P, FBP, 2PGA, 3PGA, and Acetyl‐CoA were analyzed using LC‐MS. Subsequently, 10 μl of each ammonia acetate dissolved sample was injected into an Agilent 1200 system (Agilent, Santa Clara, CA, USA) and the liquid chromatography separation was performed on a Kinetex C18 100A column (Phenomenex, Torrance, CA, USA) with mobile phase A (20 mM ammonia acetate in water) and mobile phase B (methanol). Mass spectra were acquired with SCIEX 5500 mass spectrometer under positive and negative electrospray ionization with the ion spray voltage at +5500 V. Peak integration and quantitation were performed using Analyst 1.7.1 software. Calibration curves were built for the 0.1–50 μM range.

F6P, G6P, and Pyr were analyzed using GC‐MS. Subsequently, 100 μl of each sample was dried under vacuum and derivatized with 50 μl methoxyamine hydrochloride (Sigma‐Aldrich) (40 mg/ml in pyridine) for 60 min at 50°C, then with 50 μl MSTFA + 1% TMCS (Thermo, MA, USA) at 70°C for 120 min, following a 2‐h incubation at room temperature. Chromatograms were acquired using a GC‐MS system (Agilent Inc.) consisting of an Agilent 7890 gas chromatograph, an Agilent 5975 MSD and a HP 7683B autosampler. Gas chromatography was performed on a ZB‐5MS capillary column (Phenomenex). The inlet and MS interface temperatures were 250°C, and the ion source temperature was set to 230°C. An aliquot of 1 μl was injected in splitless mode. The temperature program was 5‐min isothermal heating at 70°C, followed by an oven temperature increase of 5°C min^−1^ to 310°C and a final 10 min at 310°C. The mass spectrometer was operated in positive electron impact mode (EI) at 69.9 eV ionization energy at *m/z* 30–800 scan range. (13)C6 Glucose was spiked with samples before derivatization and was used as an internal standard. Target peaks were evaluated by the Mass Hunter Quantitative Analysis B.08.00 (Agilent) software. Calibration curve was built for 0.1–50 μg/mL concentration range.

### Untargeted lipid profiling

For lipidomic analysis, samples were initially spiked with a mixture of labeled surrogate internal standards. Postprocessing extracts were analyzed using the Thermo Q‐Exactive mass spectrometer (MS) system (Bremen, Germany), as previously described (Xue *et al*., 2020). The Dionex UltiMate 3000 series HPLC system (Thermo, Germering, Germany) was used, with LC separation performed on a Thermo Accucore C18 column with mobile phase A (60% acetonitrile : 40% water with 10 mM ammonium formate and 0.1% formic acid) and mobile phase B (90% isopropanol : 10% acetonitrile with 10 mM ammonium formate and 0.1% formic acid). The injection volume was 10 μl. Mass spectra were acquired under both positive and negative electrospray ionization. Full scan mass spectrum resolution was set to 70 000 with a scan range of *m*/*z* 230–1600. For the MS/MS scan, the mass spectrum resolution was set to 17 500. All the LC‐MS raw data files were processed using MS‐DIAL v.4.90 software for data collection, peak detection, alignment, adduct, and identification (Tsugawa *et al*., 2015). Compounds were annotated by *m/z* and MS/MS spectra against the LipidBlast mass spectra database (Kind *et al*., 2013).

### Statistical analysis

The differences between the two groups were determined using the two‐tailed Student's *t*‐test with equal variance. The differences among multiple groups were assessed using one‐way ANOVA followed by multiple comparison tests (Fisher's least significant difference method). ANOVA statistical analysis was performed using Origin 2022 statistical software (OriginLab Corp., Northampton, MA, USA).

## Results

### Overexpressing *PPT1* in Col‐0 wild‐type leaves fails to improve TFA levels

**Fig. 1:**
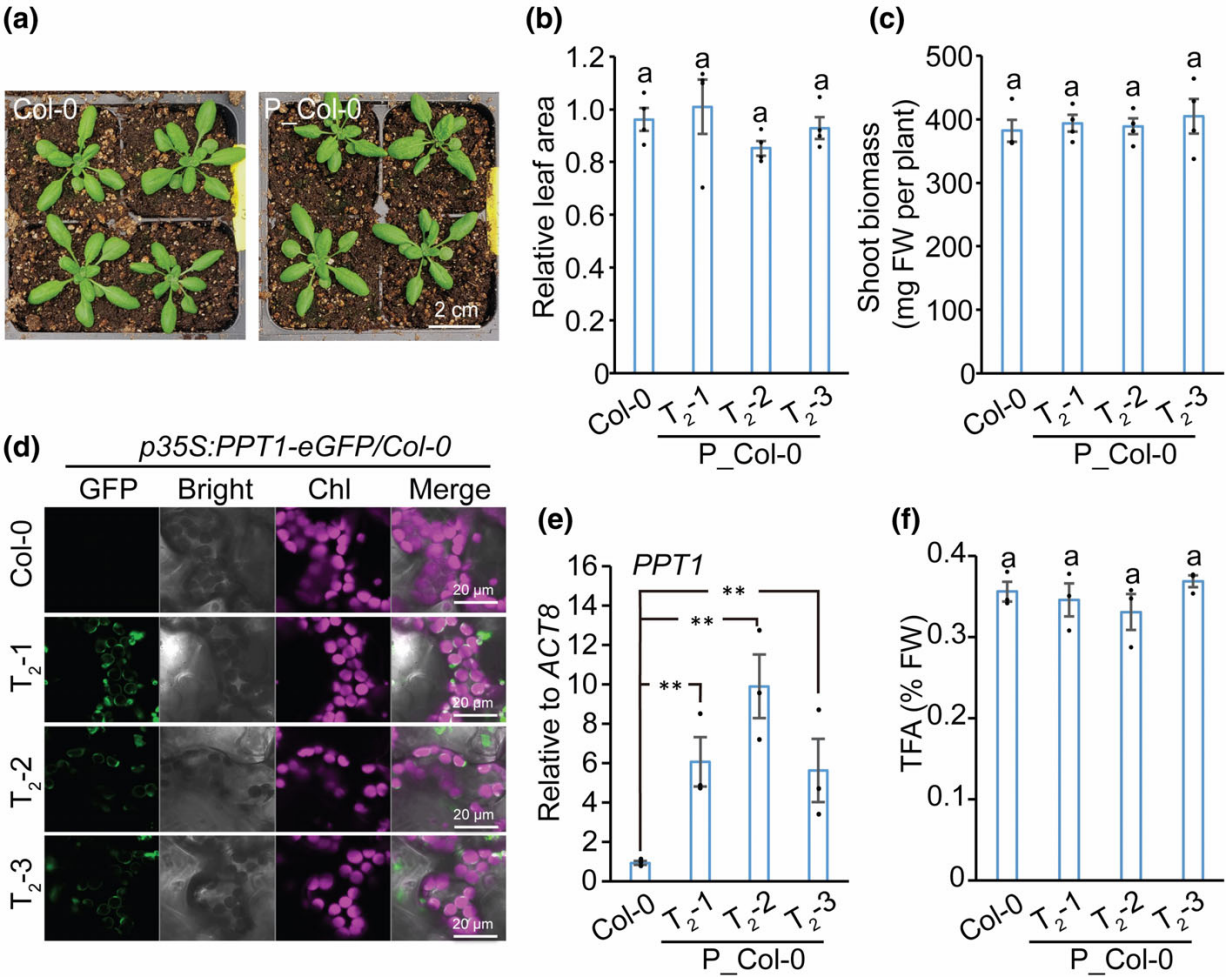
Overexpressing phospho*enol*pyruvate/phosphate translocator (*PPT1*) in the chloroplast fails to improve total fatty acids (TFA) levels in *Arabidopsis thaliana* Col‐0. (a) Phenotypical comparisons between Col‐0 and overexpressing *PPT1* in Col‐0 at 21 d post‐germination (DPG). (b, c) No significant differences observed in both leaf area (b) and shoot biomass (c) between Col‐0 and overexpressing *PPT1* in Col‐0 at 21 DPG. (d) Confocal images showing PPT1‐GFP accumulation at the chloroplast periphery in *p35S:PPT1‐eGFP* lines at 21 DPG. GFP and Chl signals were detected using 488 and 633 nm excitation, respectively. (e) Reverse transcription‐quantitative polymerase chain reaction analysis of *PPT1* expression in leaves of P_Col‐0. Data were normalized to the *ACT8* housekeeping gene using the comparative *CT* method (2^−Δ*CT*^). Means ± SE, *n* = 3; **, *P* < 0.01 by Student's *t*‐test. (f) TFA was quantified from leaves (21 DPG) (means ± SE, *n* = 4). Leaves were collected at the end of the light stage. Statistical significance assessed by one‐way ANOVA with Fisher's least significant difference (*P* < 0.05); different letters indicate significant differences.

We first overexpressed *PPT1* in the Col‐0 wild‐type and assessed the phenotypic impact of *PPT1* overexpression on vegetative growth by comparing plants at 21 DPG, a time point that minimizes the influence of reproductive growth while allowing sufficient vegetative development. Overexpression of *PPT1* in the Col‐0 (designated P_Col‐0) did not produce any visible morphological (Fig. 1a,b) or shoot biomass weight (Fig. 1c) differences compared to the untransformed control. Transgenic T_1_ and T_2_ lines were screened by confocal microscopy to verify localization. Three independent T_2_ lines displaying clear plastidial targeting were selected for further analysis. In these lines, the PPT1‐eGFP fusion protein (green signal) localized to the periphery of chloroplasts, which were identified by magenta autofluorescence (Fig. 1d). This pattern is consistent with the previously reported chloroplast envelope localization of PPT1 (Knappe *et al*., 2003). RT‐qPCR analysis confirmed successful overexpression, with all three transgenic lines showing significantly elevated PPT1 transcript levels relative to Col‐0 (Fig. 1e). Subsequently, TFA content was measured by GC‐FID. Overexpression of *PPT1* in the wild‐type background did not lead to a significant increase in TFA accumulation (Fig. 1f). This result suggests that under normal sugar conditions, carbon partitioning between the cytosol and chloroplast is tightly regulated and not readily enhanced by increased plastidic PEP import alone.

### Overexpressing *PPT1* increased the TFA content in sugar‐rich leaves

The lack of increased TFA accumulation in Col‐0 upon *AtPPT1* overexpression suggested that substrate availability, rather than PPT1 level and activity itself, may constrain plastidic carbon flux. We therefore sought to establish a genetic context in which cytosolic PEP supply would be less limiting by increasing carbon input into the glycolytic pathway through elevated sugar accumulation. To this end, we focused on mutants impaired in phloem sugar export. *SWEET11* and *SWEET12* encode sucrose efflux transporters that mediate sugar export from phloem parenchyma cells into the apoplast during phloem loading, and their loss leads to increased sugar accumulation in source leaves (Chen *et al*., 2012). However, leaves of the *sweet11;12* double mutant accumulate only *c*. 2‐fold higher levels of soluble sugars, coincident with a strong (*c*. 15‐fold) upregulation of *SWEET13*, indicating substantial functional redundancy among these transporters (Chen *et al*., 2012; Xue *et al*., 2022). To further elevate leaf sugar levels, we therefore generated a *sweet11;12;13* triple mutant by crossing *sweet13* with the *sweet11;12*, thereby creating a genetic system with enhanced cytosolic carbon availability for evaluating the metabolic impact of increased plastidial PEP import.

**Fig. 2:**
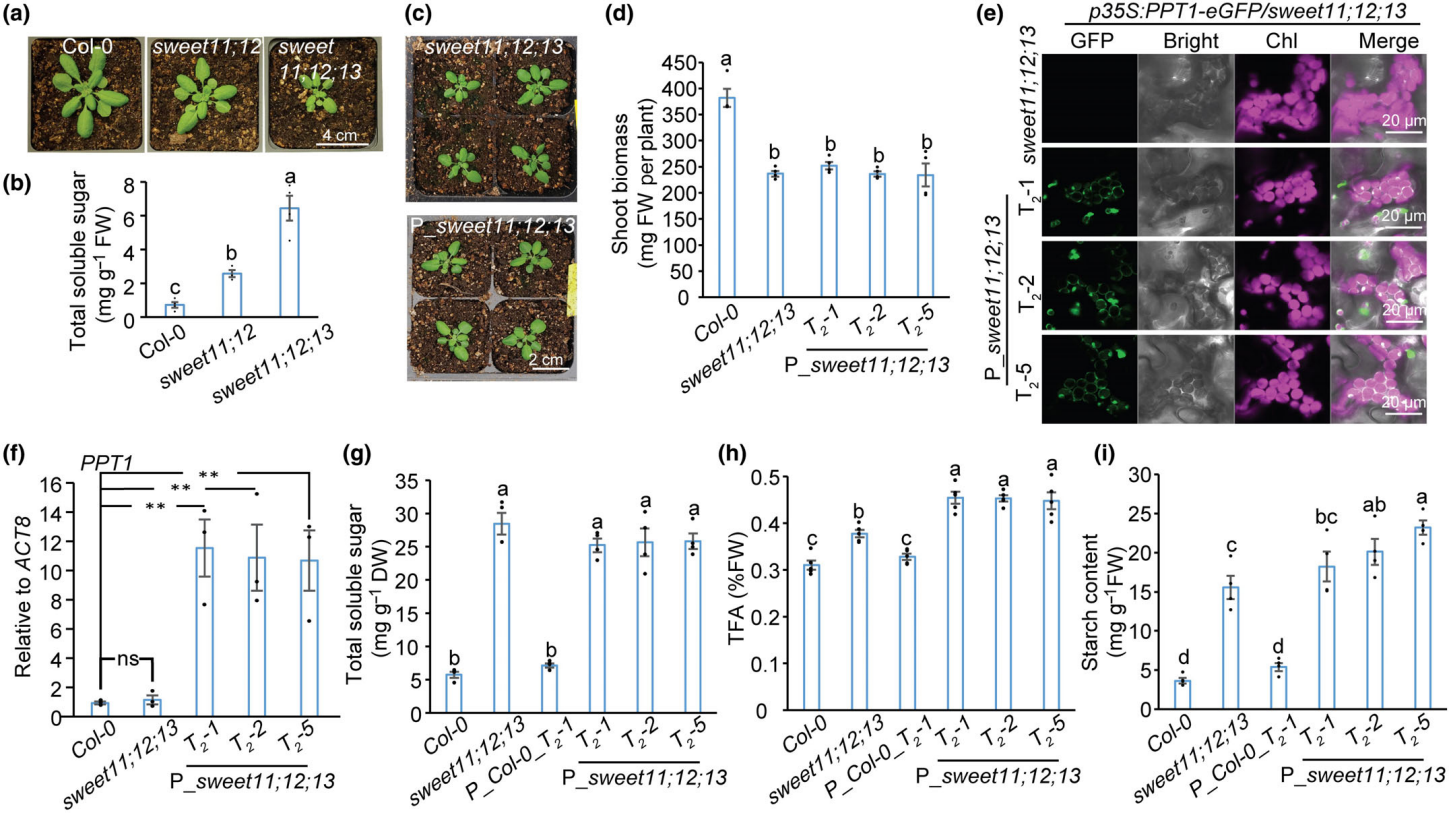
Overexpressing phospho*enol*pyruvate/phosphate translocator (*PPT1*) increased total fatty actid (TFA) content in sugar‐rich Arabidopsis mutant leaves. (a) Phenotypical comparisons among Col‐0, *sweet11;12*, and *sweet11;12;13* at 25 d post‐germination (DPG). (b) Total soluble sugar was quantified from leaves (25 DPG) of Col‐0, *sweet11;12*, and *sweet11;12;13* (means ± SE, *n* = 4). (c) Phenotypical comparisons when overexpressing *PPT1* in *sweet11;12;13* at 21 DPG. A representative picture is shown from one line of three tested independent lines. (d) *sweet11;12;13* is smaller than Col‐0, but no significant differences were observed in shoot biomass between *sweet11;12;13* and P_*sweet11;12;13* at 21 DPG. (e) PPT1 accumulation in the chloroplast periphery of transgenic lines carrying *p35S:PPT1‐eGFP* in *sweet11;12;13* visualized by confocal microscopy at 21 DPG. (f) Reverse transcription‐quantitative polymerase chain reaction (qPCR) analysis of *PPT1* expression in leaves of P_Col‐0 at 21 DPG. Data were normalized to the *ACT8* housekeeping gene using the comparative *CT* method (2^−Δ*CT*^). Means ± SE, *n* = 3; **, *P* < 0.01 by Student's *t*‐test. Figs 1(e) and 2(f) shared the same Col‐0 samples for qPCR analysis. (g**–**i) Quantification of soluble sugars (g), total fatty acids (h), and starch (i) in 21 DPG leaves (means ± SE, *n* = 4 for g and i, *n* = 5 for h). Samples were collected at the end of the light stage. Statistical significance was assessed by one‐way ANOVA and Fisher's least significant difference test (*P* < 0.05); different letters indicate significant differences.

Compared with the *sweet11;12* mutant, the *sweet11;12;13* triple mutant is more severely stunted, consistent with further impaired phloem loading (Fig. 2a). Quantification of leaf sugars showed *c*. 6‐fold higher in *sweet11;12;13* relative to Col‐0 and *c*. 2.5‐fold higher relative to *sweet11;12* (Fig. 2b). We then overexpressed *PPT1* in the *sweet11;12;13 mutant*, which did not cause any morphological (Fig. 2c) or shoot biomass weight (Fig. 2d) alterations compared to the *sweet11;12;13* control at 21 DPG. Three independent, GFP‐positive T_2_ lines were selected for subsequent analysis. Confocal microscopy revealed that PPT1‐eGFP localized to the chloroplast envelope in these lines, consistent with the localization observed in the P_Col‐0 background (Figs 1c, 2e). RT‐qPCR confirmed successful overexpression of *PPT1*, with all three transgenic lines (P_*sweet11;12;13*) showing significantly higher *PPT1* transcript levels than Col‐0, whereas transcript levels did not differ significantly between the untransformed *sweet11;12;13* mutant and Col‐0 (Fig. 2f).

We next quantified soluble sugar content by HPLC. No significant differences were found among the three independent P_Col‐0 lines (Fig. S1). Therefore, one representative line (P_Col‐0 T_2_‐1) was used for all further comparisons with P_*sweet11;12;13* unless otherwise specified. Overexpression of *PPT1* did not significantly alter this elevated sugar level, although there was a slight downward trend (Fig. 2g). Analysis of TFA content revealed that the *sweet11;12;13* mutant itself exhibited a 21.9% increase in TFA relative to Col‐0. Strikingly, the P_*sweet11;12;13* lines showed a 44.5–46.5% increase in TFA over Col‐0 and an 18.5–20.1% increase over the untransformed *sweet11;12;13* mutant (Fig. 2h). Analysis of starch content discovered that the *sweet11;12;13* mutant accumulated *c*. 3‐fold more starch relative to Col‐0, and two of the three P_*sweet11;12;13* lines showed a further significant increase in starch over the untransformed *sweet11;12;13* mutant, whereas no significant differences in starch content were observed in P_Col‐0 relative to Col‐0 (Fig. 2i).

To determine whether the developmental stage influences our strategy on TFA accumulation, we monitored rosette leaf development up to 21 DPG. At this stage, both the *sweet11;12;13* mutant and Col‐0 wild‐type plants had developed a total of eight true leaves (Fig. S2a). These leaves were grouped into four based on age: 1&2 (oldest), 3&4, 5&6, and 7&8 (youngest). Analysis of soluble sugar content revealed a clear gradient pattern in *sweet11;12;13*, with older leaves progressively accumulating higher sugar levels than younger ones (leaves 1&2 > 3&4 > 5&6 > 7&8) (Fig. S2b). This gradient is consistent with the established roles of SWEET11/12/13 in phloem loading in source leaves. For TFA content, significant differences were observed only in the older, sugar‐rich leaves (1&2 and 3&4). In these groups, *sweet11;12;13* exhibited higher TFA levels than Col‐0, and overexpression of *PPT1* in the *sweet11;12;13* background further enhanced TFA content in these leaves (Fig. S2c). This enhancing effect was attenuated in leaves 5&6 and absent entirely in the youngest leaves (7&8). Thus, leaves 3&4 are being used for all subsequent analyses.

Together, these results demonstrate that enhanced plastidic PEP import via *PPT1* overexpression effectively promotes TFA biosynthesis, but only under conditions of elevated sugar availability, consistent with a requirement for abundant cytosolic carbon supply to support increased flux into plastidial TFA biosynthesis.

### Overexpressing *PPT1* demonstrates a coordinated lipid metabolic shift in the sugar‐rich background

**Fig. 3:**
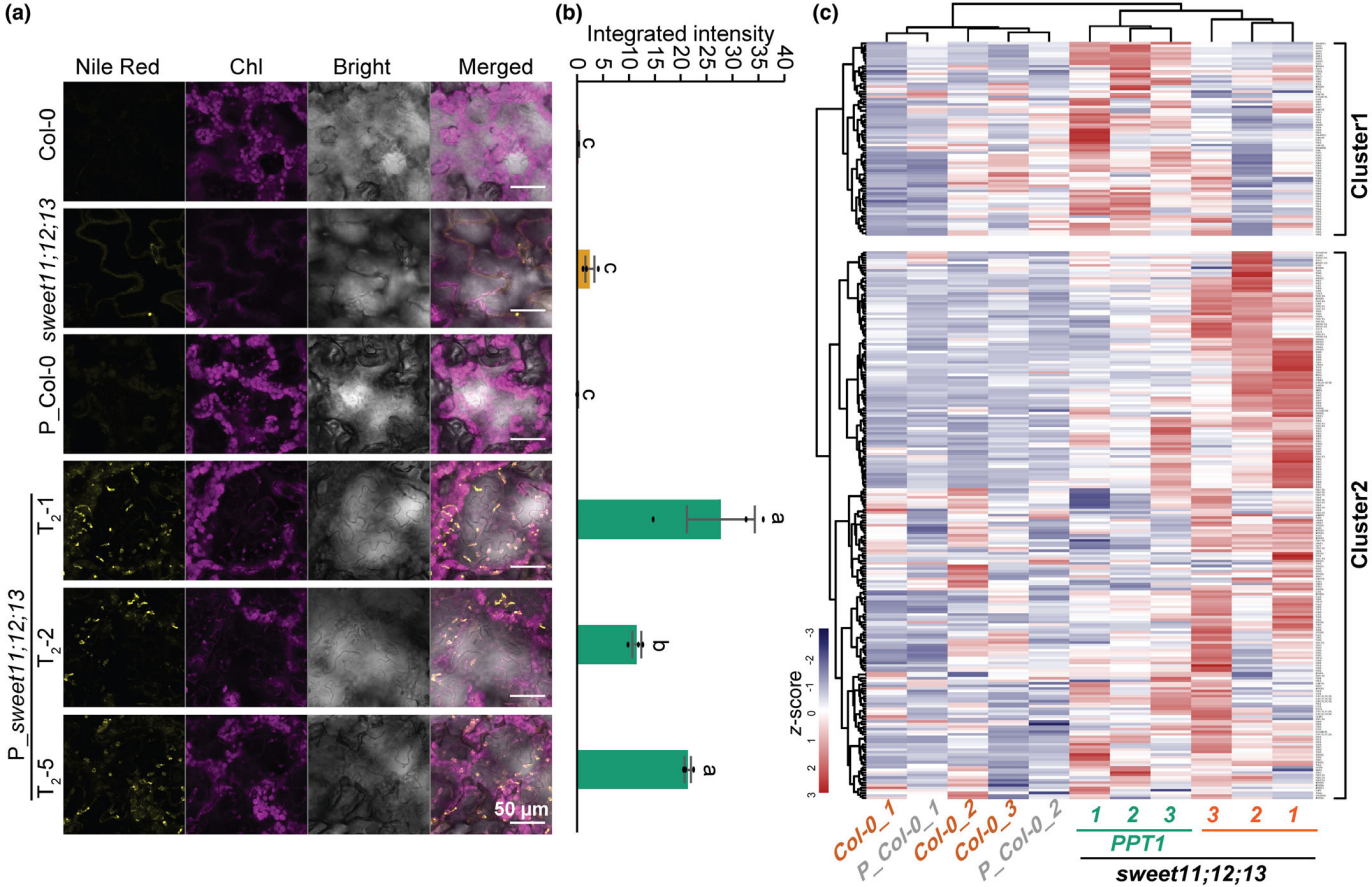
Overexpressing phospho*enol*pyruvate/phosphate translocator (*PPT1*) increased lipid accumulation in sugar‐rich Arabidopsis mutant leaves. (a) Lipid droplets (LDs) visualized by confocal microscopy after Nile Red staining. Representative images shown from one of three biological repeats. (b) Nile Red fluorescence signals quantified using ImageJ (means ± SE, *N* = 3). Statistical differences determined by one‐way ANOVA with Fisher's least significant difference test (*P* < 0.05); different letters indicate significance. (c) Lipid species profiling of various genotypes using a heatmap with hierarchical clustering. The z‐score transformed intensity values for each lipid species were plotted (red represents the highest intensity, and blue represents the lowest intensity). It should be noted that, due to outlier removal, P_Col‐0 had only two biological replicates; therefore, some trends should be interpreted with caution.

To determine whether the increase in TFA led to the formation of storage lipids, we visualized LDs in leaf tissue using Nile Red staining and confocal microscopy (Fig. 3a). LDs are dynamic organelles that store neutral lipids, primarily TAGs and sterol esters, and are surrounded by a phospholipid monolayer (Chapman *et al*., 2012). They serve as reservoirs of energy and carbon, and their abundance reflects the cellular capacity for lipid storage. LDs were clearly abundant in all three P_*sweet11;12;13* lines, whereas they were absent in both Col‐0 and P_Col‐0. Only sporadic staining spots were observed in the untransformed *sweet11;12;13* mutant compared with all controls, consistent with the observation of oleosome accumulation in the silencing mutant of potato SUT1 (Schulz *et al*., 1998), which pairs with SWEETs for the 2‐step phloem loading. Quantification confirmed a significantly higher number of LDs in the P_*sweet11;12;13* lines (Fig. 3b). To facilitate subsequent metabolic analyses, one representative transgenic line from each background (P_Col‐0 T_2_‐1 and P_*sweet11;12;13* T_2_‐2) was selected.

We next performed an untargeted global lipidomic analysis to profile changes in lipid species (Fig. 3c; Dataset S1). The results revealed an overall increase in lipids in *sweet11;12;13* compared with Col‐0, forming two distinct vertical clusters. Cluster 1 included several phosphatidylcholine (PC) and phosphatidylethanolamine (PE) species (e.g. PC(37 : 3), PC(35 : 0), PE(45 : 2)) and esterified lipid species that form the hydrophobic core of LDs, such as cinnamyl esters, syringyl esters, and zylgucosyldiacylglycerol esters. These species were higher in P_*sweet11;12;13* than in other backgrounds, indicating increased LD formation and neutral lipid storage. Cluster 2 included membrane‐associated lipids, such as phosphatidylserine and digalactosyldiacylglycerol, along with selected diacylglycerols and TAG. These species were generally lower in P_*sweet11;12;13* compared with other genotypes, suggesting a partial reallocation of carbon from structural membrane lipids toward the enhanced accumulation of neutral lipids in LDs. Some lipids showed a graded response across genotypes following the pattern: *sweet11;12;13* > P_*sweet11;12;13* > Col‐0 (e.g. PA(35 : 3), PA(35 : 2)), indicating a partial rebalancing of membrane lipid homeostasis. Collectively, these data indicate a trend toward increased accumulation of specific lipid species in *P_sweet11;12;13*, consistent with the observed increase in LD formation.

### Overexpressing *PPT1* accelerates carbon flow between cytosol and chloroplast in the sugar‐rich background

**Fig. 4:**
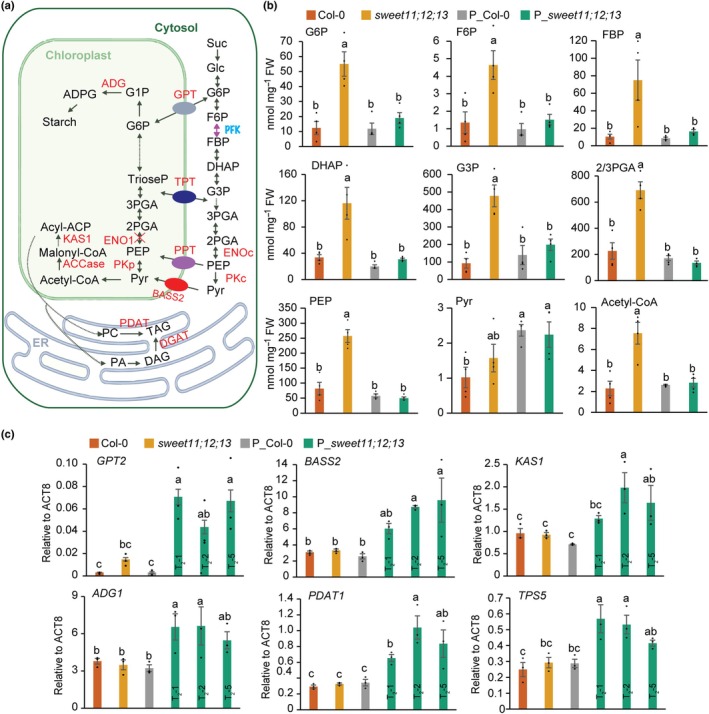
Overexpressing phospho*enol*pyruvate/phosphate translocator (*PPT1*) accelerates carbon flow between the cytosol and the chloroplast in sugar‐rich Arabidopsis mutant leaves. (a) Schematic representations of carbon flow from glycolysis to triacylglycerol synthesis. Intermediate metabolites (black) were quantified via LC‐MS or GC‐MS and the responsible genes (red) encoding the illustrated enzymes or transporters were analyzed using Reverse transcription‐quantitative polymerase chain reaction (RT‐qPCR). (b) Targeted metabolite quantifications of various genotypes such as PEP, DHAP, G3P, FBP, 2PGA, 3PGA, and Acetyl‐CoA were analyzed using LC‐MS; F6P, G6P, and Pyr were analyzed using GC‐MS from mature leaf (at 21 d post‐germination (DPG), the end of the light stage). Means ± SE, *n* = 3. (c) RT‐qPCR of some key enzymes and transporters highlighted in red of (a). Data normalized to *ACT8* using 2^−Δ*CT*^ method. RNA from leaves (21 DPG, end of light stage); means ± SE, *n* = 3. For (b, c), statistical differences determined by one‐way ANOVA with Fisher's least significant difference test (*P* < 0.05); different letters indicate significance.

Guided by the canonical carbon flow from glycolysis to starch, fatty acids, and TAG biosynthesis (Fig. 4a), we quantified key pathway intermediates via targeted LC‐MS or GC‐MS. Notably, almost all detected glycolytic intermediates were significantly elevated in *sweet11;12;13* but were restored to near wild‐type (Col‐0) levels in P_*sweet11;12;13* (Fig. 4b). Pyruvate represented a notable exception, as its levels were increased in both P_Col‐0 and P_*sweet11;12;13*, consistent with its central and multifaceted role in cellular metabolism. Together, these metabolic profiles indicate that *PPT1* overexpression enhances carbon flux from cytosolic glycolysis into plastidial metabolism specifically under sugar‐rich conditions.

To identify the molecular factors associated with the increased TFA accumulation, we analyzed the expression of key enzymes and transporters (highlighted in red in Fig. 4a) by RT‐qPCR (Fig. 4c). Transcript levels of the glucose‐6‐phosphate/phosphate translocator (*GPT2*) and the plastidic pyruvate transporter (*BASS2*) were significantly upregulated in all P_*sweet11;12;13* lines, indicating enhanced import of their substrates into plastids (Fig. 4c). Consistent with increased plastidial supply of glucose‐6‐phosphate, expression of *ADG1* (encoding a key enzyme in starch synthesis) was also elevated, corroborating the observed increase in starch content. Furthermore, *KAS1* (involved in FA elongation) was significantly upregulated, aligning with the observed increase in TFA content. TAG can be synthesized via either diacylglycerol:acyl‐CoA acyltransferase (DGAT) or phospholipid:diacylglycerol acyltransferase (PDAT), which use acyl‐CoA or phospholipids as acyl donors, respectively (Xu & Shanklin, 2016). Notably, expression of *PDAT1* was significantly induced in P_*sweet11;12;13* (Fig. 4c), whereas *DGAT1* and *DGAT2* levels remained unchanged (Fig. S3). Intriguingly, *TPS5*, encoding a class II trehalose‐6‐phosphate synthase that has been reported to lack enzymatic activity, was also markedly upregulated. By contrast, the expression of other related genes, including *GPT1*, the triose phosphate/phosphate translocator (*TPT*), cytosolic enolase (ENOc/LOS2), plastidic pyruvate kinase (*PKPβ1*), *TT4* (chalcone synthase), *BCCP2* (biotin carboxyl carrier protein), and the transcription factor *WRI1*, did not differ significantly among the genotypes (Fig. S3).

### Transcriptomic analysis reveals coordinated responses to *PPT1* overexpression under sugar‐rich conditions

To gain a global view of molecular responses to *PPT1* overexpression, we performed RNA sequencing (RNA‐seq) across all genotypes (Dataset S2). PCA showed tight clustering of biological replicates. Genotypic differences were primarily separated along PC2, which distinguished the Col‐0 and *sweet11;12;13* backgrounds, while the effect of *PPT1* overexpression was captured along PC1 (Fig. S4a). As expected, *SWEET11*, *12*, and *13* transcripts were significantly reduced in *sweet11;12;13* (Fig. S4b). In total, we identified 2382 DEGs across all pairwise comparisons (Dataset S2), providing a comprehensive framework for dissecting transcriptional changes associated with enhanced plastidial PEP import under sugar‐rich conditions.

**Fig. 5:**
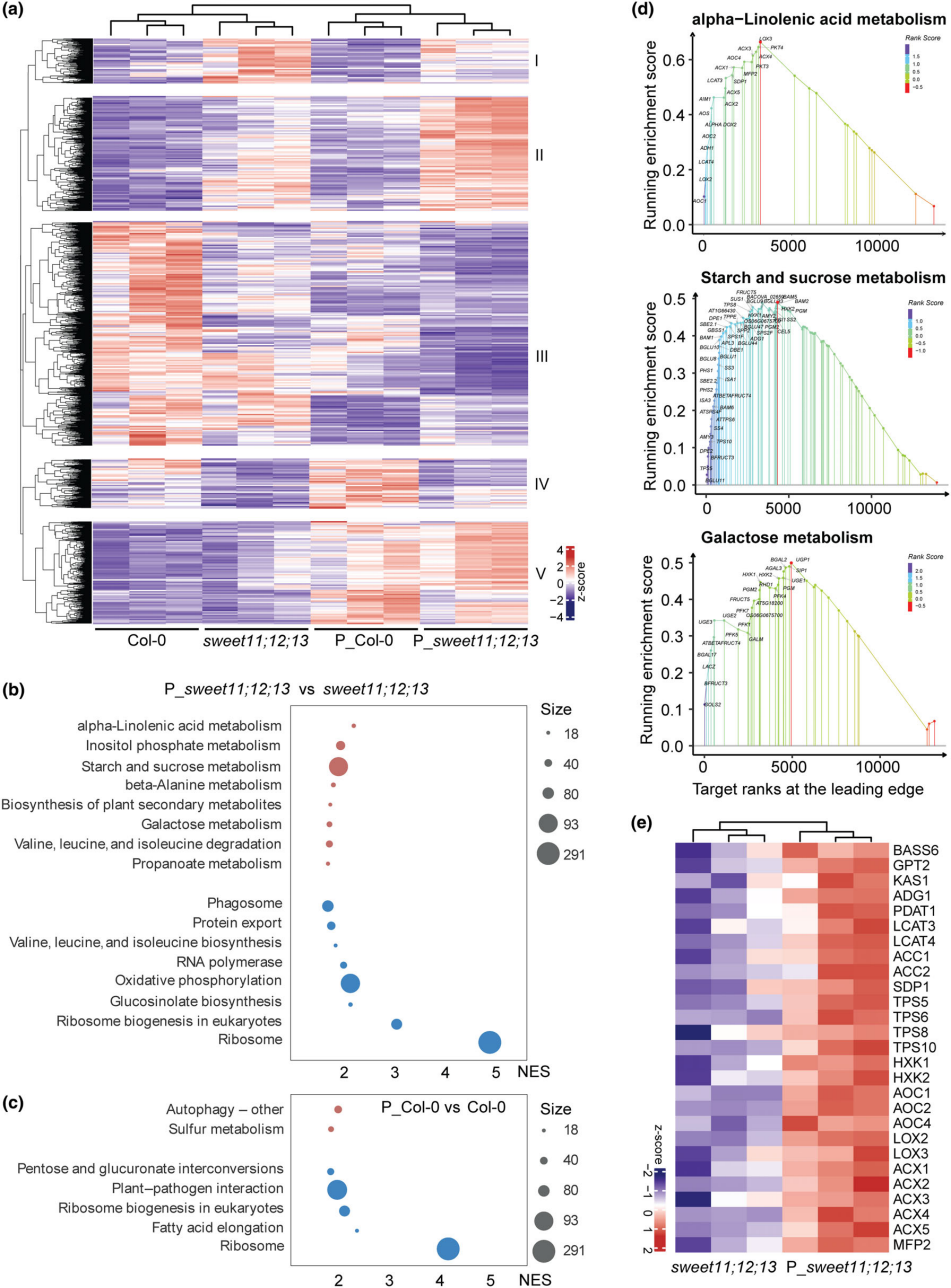
Transcriptomic analysis reveals coordinated responses to phospho*enol*pyruvate/phosphate translocator (*PPT1*) overexpression in sugar‐rich Arabidopsis mutant leaves. (a) Heatmap with hierarchical clustering of differentially expressed genes (DEGs) based on *z*‐score‐transformed TPM values of each DEG (purple, low; red, high expression). (b, c) Gene set enrichment analysis (GSEA) for *PPT1* overexpression under sugar‐rich (b) and normal (c) conditions. Kyoto Encyclopedia of Genes and Genomes enrichment analysis was performed with a flase discovery rate *q*‐value cutoff of 0.05. Negative normalized enrichment scores (NES) values were converted to absolute values (blue dots) to display alongside positive NES (red dots) on the same y‐axis. (d) Positional enrichment plots for key gene sets (α‐linolenic acid, starch/sucrose, galactose metabolism). Rank score (signal : noise ratio) indicated by color (purple, high; red, low). (e) Heatmap of candidate genes with column clustering; *z*‐score‐transformed TPM values (purple, low; red, high).

Hierarchical clustering of these DEGs produced a heatmap in which the three biological replicates of each genotype clustered tightly together, and *PPT1*‐overexpressing lines were clearly separated from control lines (Fig. 5a). Row‐wise clustering revealed five major gene clusters, with cluster 2 genes highly enriched in P_*sweet11;12;13*. KEGG pathway enrichment analysis indicated significant pathway enrichment in clusters 1, 2, and 3, whereas no significant enrichment was detected for clusters 4 or 5. Notably, pathways enriched in cluster 2 included alpha‐linolenic acid metabolism and starch and sucrose metabolism, both of which are directly relevant to lipid accumulation and carbon partitioning (Fig. S5).

To systematically assess how *PPT1* overexpression reshapes transcriptional programs under contrasting sugar regimes, we performed GSEA. In the sugar‐rich *sweet11;12;13* background (Fig. 5b), *PPT1* overexpression led to significant enrichment of gene sets associated with metabolism (e.g. alpha‐linolenic acid metabolism), carbohydrate metabolism (starch, sucrose, galactose, and inositol phosphate metabolism), and amino acid metabolism (beta‐alanine and branched‐chain amino acid (BCAA) degradation). Conversely, gene sets involved in translation (ribosome biogenesis), transcription (RNA polymerase), oxidative phosphorylation, BCAA synthesis, and phagosome‐related transport and catabolism were significantly downregulated. By contrast, *PPT1* overexpression in the normal‐sugar Col‐0 background produced a markedly different transcriptional response (Fig. 5c). Only a limited number of gene sets were upregulated, including sulfur metabolism and autophagy, whereas gene sets associated with translation, fatty acid elongation, plant–pathogen interactions, and pentose and glucuronate interconversions were downregulated.

To identify key drivers within the enriched pathways under sugar‐rich conditions, we examined individual gene expression patterns within selected gene sets (Fig. 5d). In the alpha‐linolenic acid metabolism set, multiple genes encoding enzymes critical for jasmonic acid (JA) biosynthesis (*AOC*, *LOX*, *ACX*, and *MFP*) (Bell *et al*., 1995; Schilmiller *et al*., 2007; Stenzel *et al*., 2012) were strongly upregulated (Fig. 5d,e). Within the starch and sucrose metabolism set, several class II trehalose‐6‐phosphate synthase genes (*TPS5*, *6*, *8* and *10*) (Ramon *et al*., 2009), exhibited pronounced induction. In addition, *HXK1* and *HXK2*, which encode hexose kinase that function both in glycolytic carbon flux and as glucose sensors, were also highly upregulated (Fig. 5d,e). We further confirmed the elevated expression of the aforementioned candidate genes, along with other relevant genes identified by RT‐qPCR (e.g. *BASS6*, *LCATs*, *ACCs*, and *SDP1*), specifically in the P_*sweet11;12;13* lines (Fig. 5e).

### PFK activity is elevated by PPT1 overexpression

**Fig. 6:**
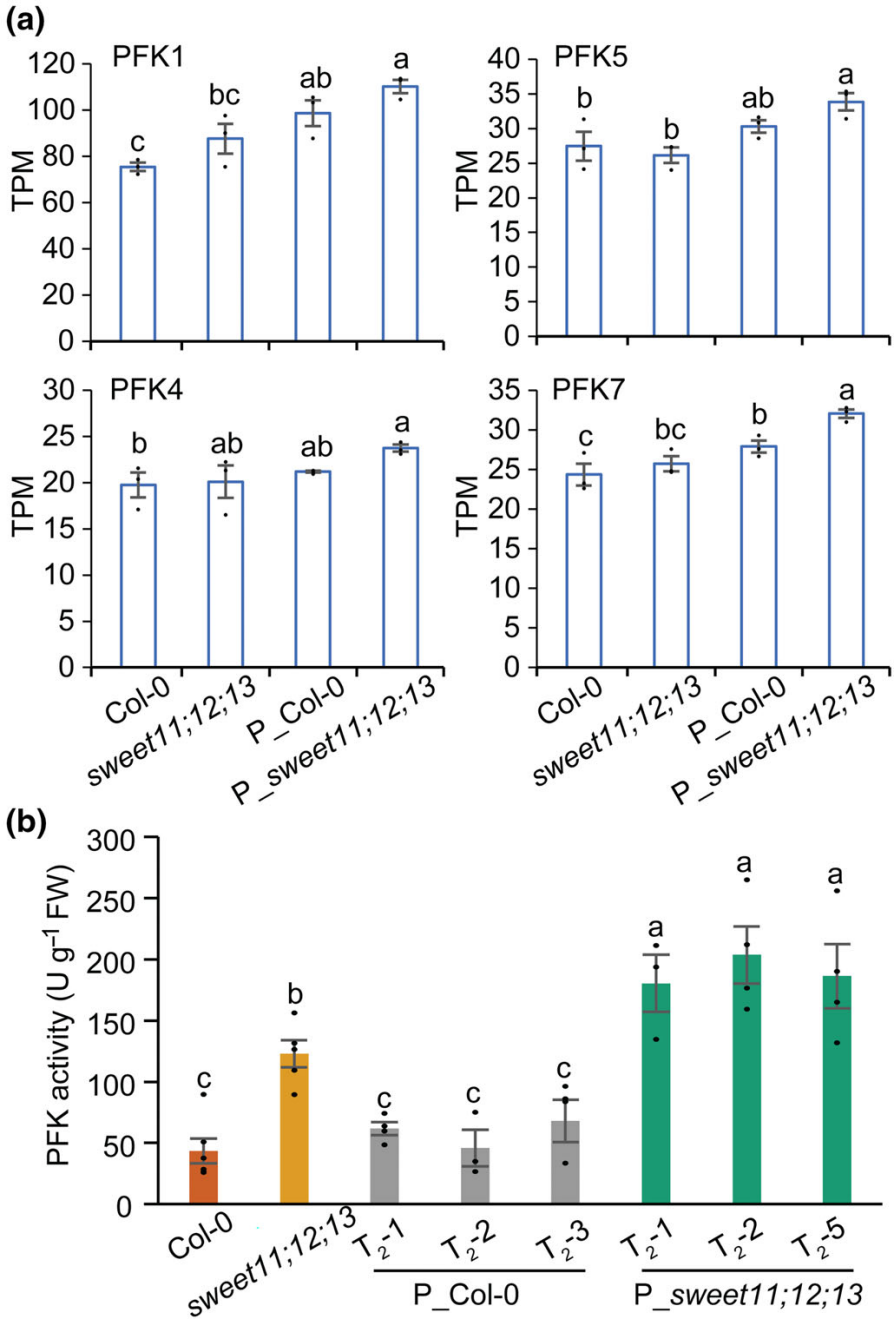
Phosphofructokinase (PFK) is elevated upon phospho*enol*pyruvate/phosphate translocator overexpression in sugar‐rich Arabidopsis mutant *sweet11;12;113*. (a) Transcript levels (TPM) of various PFK genes retrieved from the RNA‐seq dataset (means ± SE, *n* = 3). (b) PFK enzyme activity quantification from various genotypes at 21 d post‐germination (DPG) (means ± SE, *n* = 3–6). Statistical differences determined by one‐way ANOVA with Fisher's least significant difference test (*P* < 0.05); different letters indicate significance.

Within the galactose metabolism gene set, three of the seven Arabidopsis *PFK* genes (*PFK1*, *PFK5*, and *PFK7*) were significantly upregulated in P_*sweet11;12;13* compared with the *sweet11;12;13* control (Figs 5d, 6a). *PFK4* transcript abundance also showed a modest increasing trend, though not significant. In addition, *PFK1* and *PFK7* were significantly upregulated in P_Col‐0 relative to the Col‐0 control (Fig. 6a), while no significant differences were observed between Col‐0 and *sweet11;12;13* alone. Given that PFK is a key enzyme in glycolysis and is subject to allosteric inhibition by PEP in different organisms (Kelly & Latzko, 1981; Kimmel & Reinhart, 2000), we hypothesized that enhanced plastidial PEP import via *PPT1* overexpression could alleviate PEP‐mediated inhibition, particularly in the PEP‐accumulating *sweet11;12;13* mutant (Fig. 4b). To test this possibility, we measured PFK enzymatic activity. Consistent with the transcriptional data, PFK activity was significantly elevated in all three P_*sweet11;12;13* lines (Fig. 6b). This enhancement was specific to the sugar‐rich background, as no significant increase was detected between P_Col‐0 and Col‐0. Interestingly, PFK activity was also higher in the *sweet11;12;13* mutant itself compared to Col‐0 (Fig. 6b). Collectively, these changes in PFK activity align with the observed increases in TFA accumulation across genotypes. The results indicate that glycolytic flux is stimulated in sugar‐rich conditions and can be further amplified by increasing plastidial PEP import through *PPT1* overexpression.

## Discussion

In this study, we constitutively overexpressed *AtPPT1* in Arabidopsis. No significant increase in leaf TFA content was observed in the Col‐0 background (Fig. 1f), indicating that *PPT1* does not limit flux under normal conditions. This finding suggests that PEP partitioning is tightly regulated to balance the metabolic demands of both cytosolic and plastidic compartments. Such regulation is likely facilitated by the well‐documented metabolic coordination between chloroplasts and mitochondria, including the reported substrate channeling of glycolytic intermediates like phosphoglycerate (PGA) and PEP between these organelles (Zhang *et al*., 2020). By contrast, *PPT1* overexpression in the sugar‐rich *sweet11;12;13* mutant significantly enhanced both TFA content accumulation (Fig. 2h). These results imply that under conditions of carbon excess, increased plastidial PEP import can be accommodated to stimulate fatty acid synthesis, without compromising the cytosolic intermediates required for mitochondrial respiration. This model is further supported by our metabolomic data: glycolytic intermediates from glucose‐6‐phosphate to acetyl‐CoA were markedly elevated in *sweet11;12;13* but restored to near wild‐type levels in the P_*sweet11;12;13* lines (Fig. 4b). Previous work has shown that increasing sugar availability promotes carbon flux into FA and TAG synthesis in Arabidopsis (Zhai *et al*., 2017). Consistently, our data showed that the *sweet11;12;13* mutant itself accumulated more TFA and TAG than Col‐0, and *PPT1* overexpression provided a further significant boost (Figs 2h, 3). Notably, no visible LDs were detected in the Col‐0 background, which aligns with previous observations that vegetative tissues typically contain only trace amounts of LDs under normal growth conditions (Pyc *et al*., 2017). Under stress conditions such as senescence, drought, or fungal infection, however, LDs have been shown to accumulate in vegetative tissues (Lu *et al*., 2020). Whether *PPT1* overexpression enhances lipid synthesis specifically under such stress conditions remains an open question for future investigation.

**Fig. 7:**
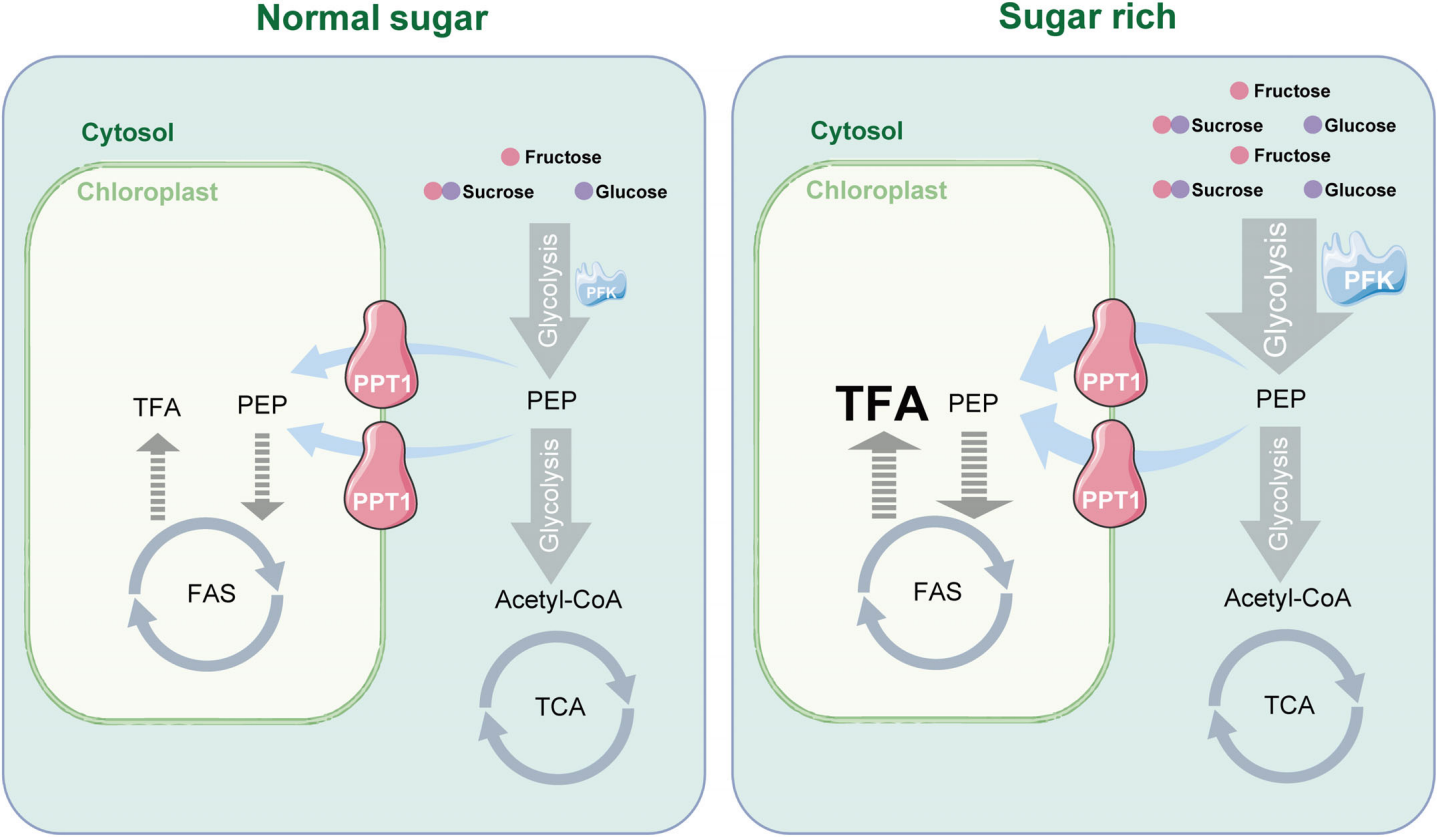
Working model of overexpressing *PPT1* on fatty acid synthesis (FAS) under contrasting sugar accumulation conditions in Arabidopsis leaves. Under normal sugar conditions, overexpressing *PPT1* does not affect cytosolic glycolysis nor *de novo* FAS in the chloroplast. By contrast, under sugar‐rich conditions, overexpressing *PPT1* accelerated cytosolic glycolysis, likely via elevating PFK enzyme activity, and stimulated FAS in the chloroplast. PEP, phospho*enol*pyruvate; PFK, phosphofructokinase; PPT1, phospho*enol*pyruvate/phosphate translocator; TFA, total fatty acids.

A key mechanistic insight from this work is the observed reduction in PEP coupled with increased PFK activity in P_*sweet11;12;13* lines compared with those from *sweet11;12;13*. These changes support the idea that PEP likely acts as a feedback inhibitor of PFK *in vivo*, consistent with earlier *in vitro* studies demonstrating PEP‐mediated allosteric inhibition of plant PFKs (Kelly & Latzko, 1981; Plaxton, 1996; O'Leary & Plaxton, 2020). Although this regulatory relationship has long been hypothesized, direct physiological evidence in plants has been limited. Our findings support a model in which enhanced cytosolic PEP import into plastids via *PPT1* overexpression lowers cytosolic PEP availability, thereby relieving allosteric inhibition of PFK and accelerating glycolytic flux. These findings identify plastidial PEP import as a potential regulatory node linking cytosolic glycolysis to plastidial lipid metabolism in plants. We have integrated these observations into a working model that illustrates the context‐dependent effects of *PPT1* overexpression on carbon partitioning and fatty acid synthesis (Fig. 7).

The synergistic enhancement of TFA biosynthesis by sugar accumulation and *PPT1* overexpression appears to extend beyond a simple ‘push‐pull’ mechanism. We observed a coordinated transcriptional response exclusive to the P_*sweet11;12;13* lines, evidenced by strong upregulation of *GPT2*, *BASS2*, *ADG1*, *KAS1*, *PDAT1*, and *TPS5*. RNA‐seq analysis further revealed specific induction of pathways related to starch/sucrose metabolism, α‐linolenic acid metabolism, and galactose metabolism only in the sugar‐rich background with *PPT1* overexpression. Notably, several class II trehalose‐6‐phosphate synthase genes (*TPS5*, *TPS6*, *TPS8*, and *TPS10*), which lack canonical enzymatic activity (Ramon *et al*., 2009), were highly induced. Recent evidence suggests class II TPS proteins can interact with and inhibit SnRK1 (Van Leene *et al*., 2022), a central kinase regulating energy and carbon status (Crepin & Rolland, 2019). The specific upregulation of hexokinase (HXK1/2) and JA biosynthesis genes in P_*sweet11;12;13* further points to the activation of integrated signaling pathways involving sugar sensing, growth regulation, and stress responses (Rolland *et al*., 2002; Wasternack & Song, 2017). Thus, *PPT1* overexpression in sugar‐rich tissues initiates a broad transcriptional reprogramming that integrates enhanced metabolic flux with regulatory signaling pathways.

In summary, we demonstrate that *PPT1* overexpression effectively enhances TFA synthesis specifically and storage lipid accumulation under sugar‐rich conditions. This strategy represents a complementary module that can be integrated with established lipogenic factors (e.g. *WRI1*, *DGAT1*, *OLE1*) (Khan *et al*., 2025) to optimize carbon partitioning and boost oil accumulation in vegetative tissues. Our findings provide a promising strategy for metabolic engineering aimed at improving oil yield in high‐biomass biofuel crops.

## Competing interests

None declared.

## Author contributions

L‐QC supervised the project. JW and L‐QC designed the experiments. JW, Y‐HL, XX, GB and YL performed the experiments and analyzed the data. JL generated triple mutant plants. JW and L‐QC wrote the manuscript. XX edited the manuscript.

## Disclaimer

The New Phytologist Foundation remains neutral with regard to jurisdictional claims in maps and in any institutional affiliations.

## Supporting information


**Dataset S1** Lipid species in various genotypes and clusters.

**Dataset S2** Differentially expressed gene table for various plant materials and cluster annotations.

**Fig. S1** Sugar content analysis in three independent lines of each transgenic material.
**Fig. S2** Increased fatty acid content in sugar‐accumulating mature leaves of *sweet11;12;13*.
**Fig. S3** Reverse transcription‐quantitative polymerase chain reaction analysis of genes coding for relevant enzymes in various genotypes.
**Fig. S4** PCA of transcriptomic data.
**Fig. S5** Kyoto Encyclopedia of Genes and Genomes enrichment analysis.
**Table S1** Primers used in this study.
Please note: Wiley is not responsible for the content or functionality of any Supporting Information supplied by the authors. Any queries (other than missing material) should be directed to the *New Phytologist* Central Office.

## Data Availability

The RNA‐seq raw data are available in the National Center for Biotechnology Information (NCBI) GEO database via the accession number GSE282314 (https://www.ncbi.nlm.nih.gov/geo/query/acc.cgi?acc=GSE282314). The *AtPPT1* sequence is publicly available in the NCBI database under accession number At5G33320.
