# Supplementary material for: PPT1‐mediated plastidic phospho enol pyruvate import enhances fatty acid biosynthesis in sugar‐rich tissues
Source: New Phytol. 2026 Apr 7;250(6):3900–13. doi: 10.1111/nph.71160 (PMC13193356; doi:10.1111/nph.71160)
Supplement: Supplementary file 3 — Fig. S1 Sugar content analysis in three independent lines of each transgenic material. Fig. S2 Increased fatty acid content in sugar‐accumulating mature leaves of sweet11;12;13. Fig. S3 Reverse transcription‐quantitative polymerase chain reaction analysis of genes coding for relevant enzymes in various genotypes. Fig. S4 PCA of transcriptomic data. Fig. S5 Kyoto Encyclopedia of Genes and Genomes enrichment analysis. Table S1 Primers used in this study. Please note: Wiley is not responsible for the content or functionality of any Supporting Information supplied by the authors. Any queries (other than missing material) should be directed to the New Phytologist Central Office. [file NPH-250-3900-s001.pdf]

New Phytologist

***New Phytologist* Supporting Information**

Article title: **PPT1-mediated plastidic phospho*enol*pyruvate import enhances fatty acid biosynthesis in sugar-rich tissues**

Authors: Jiang Wang, Yi-Hsuan Lin, Xueyi Xue, Gabriel Beuchat, Yaxin Li, Jiankun Li and Li-Qing Chen

Article acceptance date: 17 March 2026

The following Supporting Information is available for this article:

Fig. S1 Sugar content analysis in three independent lines of each transgenic material.

Fig. S2 Increased fatty acid content in sugar-accumulating mature leaves of *sweet11;12;13*.

Fig. S3 RT-qPCR analysis of genes coding for relevant enzymes in various genotypes.

Fig. S4 PCA analysis of transcriptomic data.

Fig. S5 KEGG enrichment analysis.

Table S1 Primers used in this study.

Dataset S1 Lipid species in various genotypes and clusters. (Separate file)

Dataset S2 Differentially expressed genes table for various plant materials and cluster annotations. (Separate file)

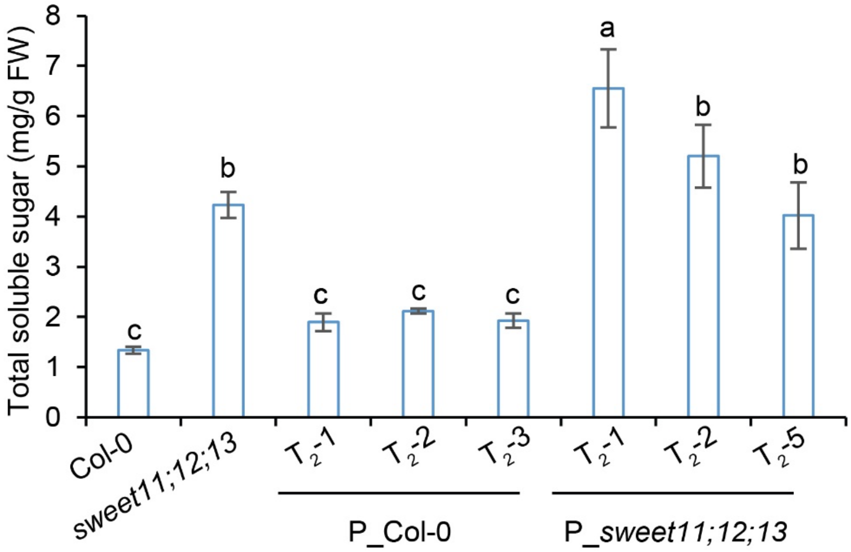

Fig. S1 Sugar content analysis in three independent lines of each transgenic material.

The content of sugar was quantified using HPLC equipped with RID. Means of four (± SE) replicates were plotted. The statistically significant differences among samples were determined using one-way ANOVA followed by multiple comparison tests (Fisher's LSD method) and were represented by different letters ($P < 0.05$).

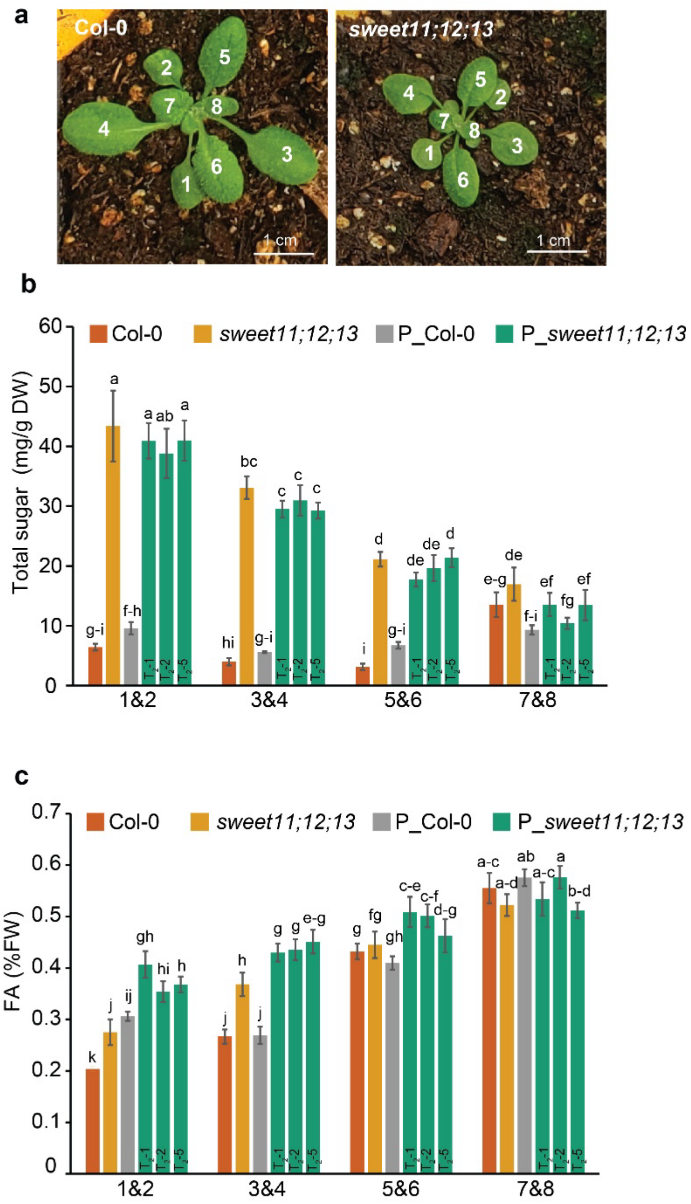

Fig. S2 Increased fatty acid content in sugar-accumulating mature leaves of *sweet11;12;13*.

(a) Leaf numbering of Col-0 and *sweet11;12;13* at 21 DPG. (b) Total soluble sugars were quantified from various leaf groups at 21 DPG using HPLC equipped with RID (means± SE, $N = 4$). (c) Total fatty acids were quantified from various leaf groups at 21 DPG (means± SE, $N = 5$). The statistically significant differences among samples were determined using one-way ANOVA followed by multiple comparison tests (Fisher's LSD method) and were represented by different letters ($P < 0.05$).

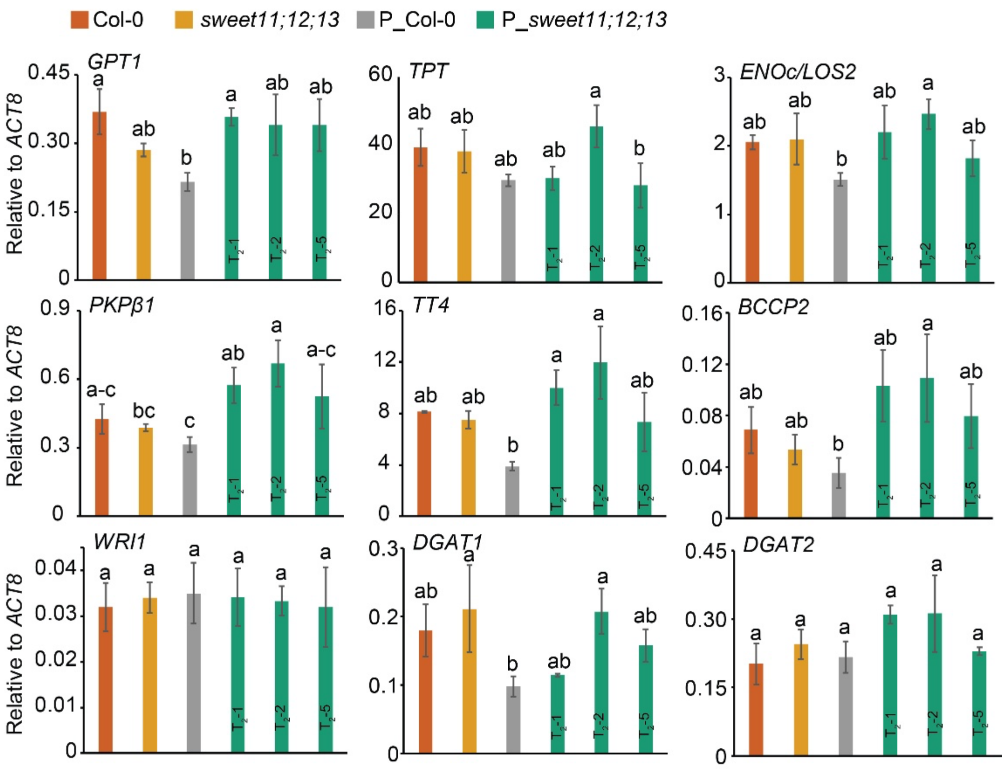

Fig. S3 RT-qPCR analysis of genes coding for relevant enzymes in various genotypes.

The expression data were normalized to the *ACT8* housekeeping gene using the comparative Ct method ($2^{-\Delta CT}$). Means (±SE) from three independent repeats were plotted. RNA was extracted from leaves 3&4 at the end of the light stage at 21 DPG. Statistically significant differences among samples were determined using one-way ANOVA followed by multiple comparison tests (Fisher's LSD method) and were represented by different letters ($P < 0.05$).

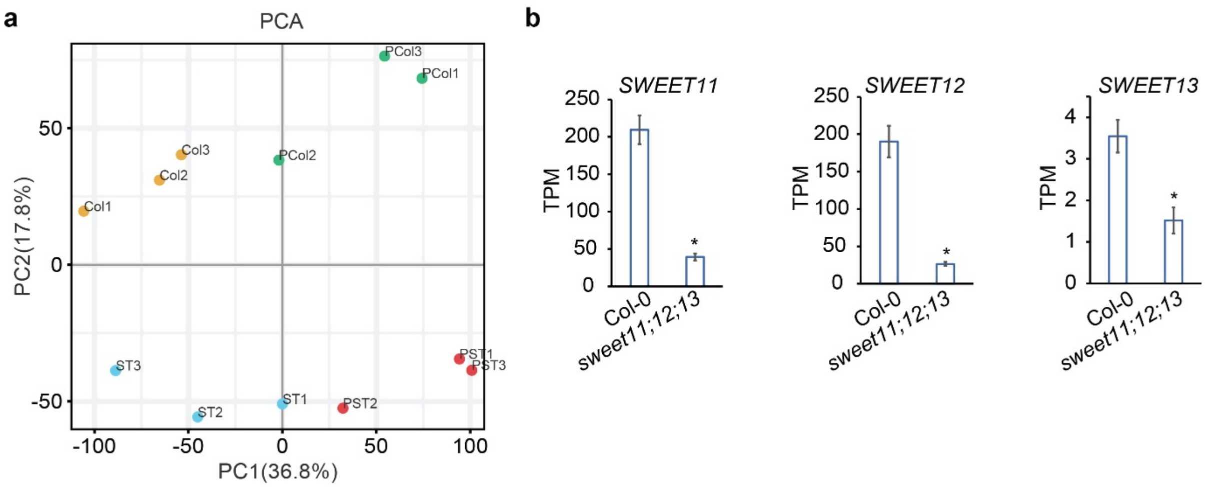

Fig. S4 PCA analysis of transcriptomic data.

(a) PCA analysis was performed with three independent repeats represented using the same color. (b) *SWEET11, 12, 13* were significantly down-regulated in *sweet11;12;13.* TPMs of *SWEET11, 12, 13* genes were retrieved from the RNA-seq dataset. Means (±SE) from three independent repeats were plotted. The statistical differences were represented by " * " using a T-test (*P*<0.05).

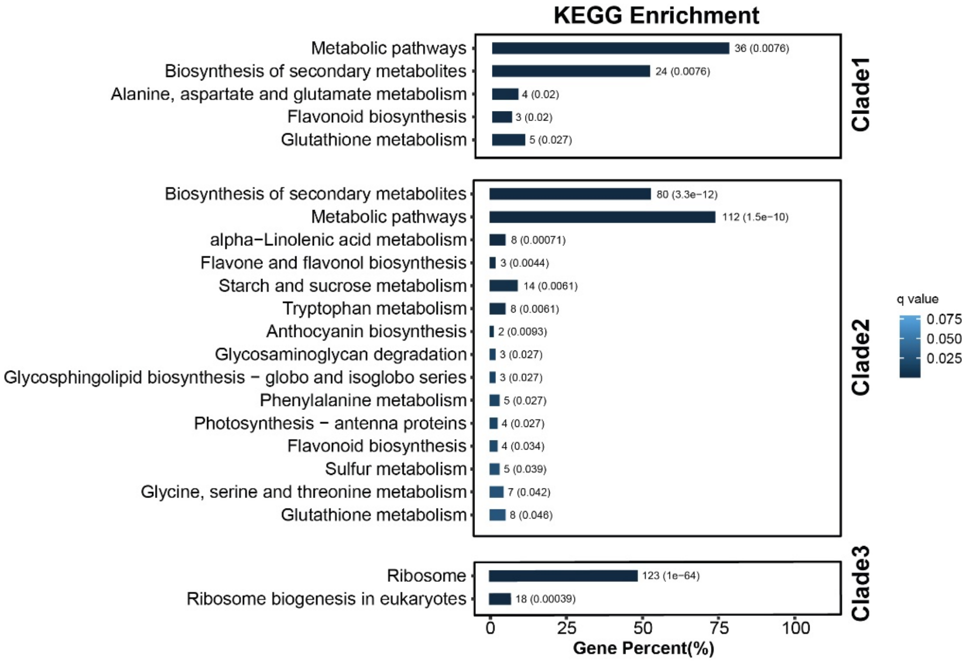

Fig. S5 KEGG enrichment analysis.

DEGs from clades 1-3 were used to perform the KEGG enrichment analysis. An adjusted *P*-value of 0.05 (q-value) was used as the cutoff.

Table S1 Primers used in this study.

#	Name	Sequences
P1	SWEET11-LP	CCGAAGAGTAATGTGACCACG
P2	SWEET11-RP	TGAAGTGGGTGCTTTTGTTTC
P3	SWEET12-LP	ATGCAGGCCAACGTTCTATAG
P4	SWEET12-RP	TCAAAGGCCAAAGCAATATACC
P5	SWEET13-LP	TACGCTATGCAAAAAGATGGC
P6	SWEET13-RP	CGACAAAAGAAGTTGGCAAAG
P7	ACT8-qF	CACTTTCCAGCAGATGTGGATC
P8	ACT8-qR	AATGCCTGGACCTGCTTCAT
P9	AtPPT1-qF	TGCAGTTCCTGAAAGTGCTG
P10	AtPPT1-qR	CCAACAGCAAACTGAACCAA
P11	GPT2-qF	CTTCGAATTTCAAGCGTGAGG
P12	GPT2-qR	TCTGCGCGGATTGTTCATC
P13	BASS2-qF	TGTTCAACCATTGGGGCTAT
P14	BASS2-qR	CCGATTAGAGGCGTCACTGT
P15	KAS1-qF	CACAATTAACAGCACCTCCAAG
P16	KAS1-qR	TGGGATAAAGCAAGAGATGGG
P17	ADG1-qF	TGGTTACTGGGAAGACATTGGT
P18	ADG1-qR	CCCTCTCCGATGACACTGTC
P19	PDAT1-qF	TGTTGCAGGGCTTTTCTCTG
P20	PDAT1-qR	TGTTGAGTCCCATGTGCG
P21	TPS5-qF	AAGAGCTTATGGAACACCTCG
P22	TPS5-qR	AGACCTTTGTTCACACCCTG
P23	GPT1-qF	TGGCTTACCTCGACGCTTTCT
P24	GPT1-qR	TACTCACCGTTGCAGCCACAT
P25	TPT-qF	TGAAACGTGTGTTCGTGATCG
P26	TPT-qR	TGCAACTCCAGCAATGGCTAT
P27	ENOc-qF	ATGGCTACTATCACCGTTGTT
P28	ENOc-qR	CTGGCCCGATGATGTTGTTC
P29	PKPβ1-qF	CTCCATACCTAACTTGCACTCC

P30	PKPβ1-qR	CTCTGCACCAAGATCACCTC
P31	TT4-qF	GGAAGTCAGCTAAGGATGGTG
P32	TT4-qR	GTCCGAAACCAAACAAGACAC
P33	BCCP2-qF	AACAGCAAAACCAACATCCG
P34	BCCP2-qR	CCCTTCTGTACCTTATCTCCAAC
P35	WRI1-qF	TCGGAAGAGTGTTTGGGAAC
P36	WRI1-qR	CAATCGCAGCCATGTCATATG
P37	DGAT1-qF	TTGGATTCTGCTGGCGTTAC
P38	DGAT1-qR	GCCTCTTCCACCACCGTTAT
P39	DGAT2-qF	TGCGCATAGCCATGGAACAG
P40	DGAT2-qR	TGGTTTACCAACGACCACATGC

Dataset S1 Lipid species in various genotypes and clusters. (Separate file)

Dataset S2 Differentially expressed genes table for various plant materials and cluster annotations. (Separate file)
